# Hidden in plain sight: a maize ‘PAL’ with aminomutase activity

**DOI:** 10.1111/tpj.71122

**Published:** 2026-09-17

**Authors:** Charlay Wood

For years, Zm00001d033286 sat in the maize reference genome annotated as a phenylalanine ammonia‐lyase (PAL). It was a reasonable designation since the enzyme genuinely has PAL activity. Here, Sakamoto and colleagues show that this annotation captures only one part of its chemistry (Sakamoto et al., 2026). The enzyme can also move the amino group of tyrosine from the α‐ to the β‐carbon, producing the non‐proteinogenic amino acid (R)‐β‐tyrosine. Renamed ZmTAM1, it combines tyrosine aminomutase (TAM), PAL and tyrosine ammonia‐lyase (TAL) activities on a single scaffold. Its product inhibits the radicle growth of *Striga hermonthica*, a parasitic weed responsible for severe losses in maize production.

For Sakamoto, the discovery reinforced ‘the importance of experimentally verifying gene function rather than relying solely on database annotations’. The rice enzyme OsTAM1 was also originally catalogued as a PAL (Yan et al., 2015), leading him to suspect that ‘there are more examples like this waiting to be discovered’.

PAL is a gateway between primary metabolism and phenylpropanoid biosynthesis (Zhang & Liu, 2015). In grasses, many PAL enzymes also accept tyrosine and are therefore designated phenylalanine/TALs, or PTALs (Barros et al., 2016). PAL and TAL reactions release a substrate's amino group as ammonia. Aminomutases use the same MIO (4‐methylideneimidazole‐5‐one) cofactor but relocate the amino group to the β‐carbon instead (Figure 1). Although TAM had long been known in bacteria, it was identified in plants only in 2015 (Christenson et al., 2003; Yan et al., 2015).

**Figure 1:**
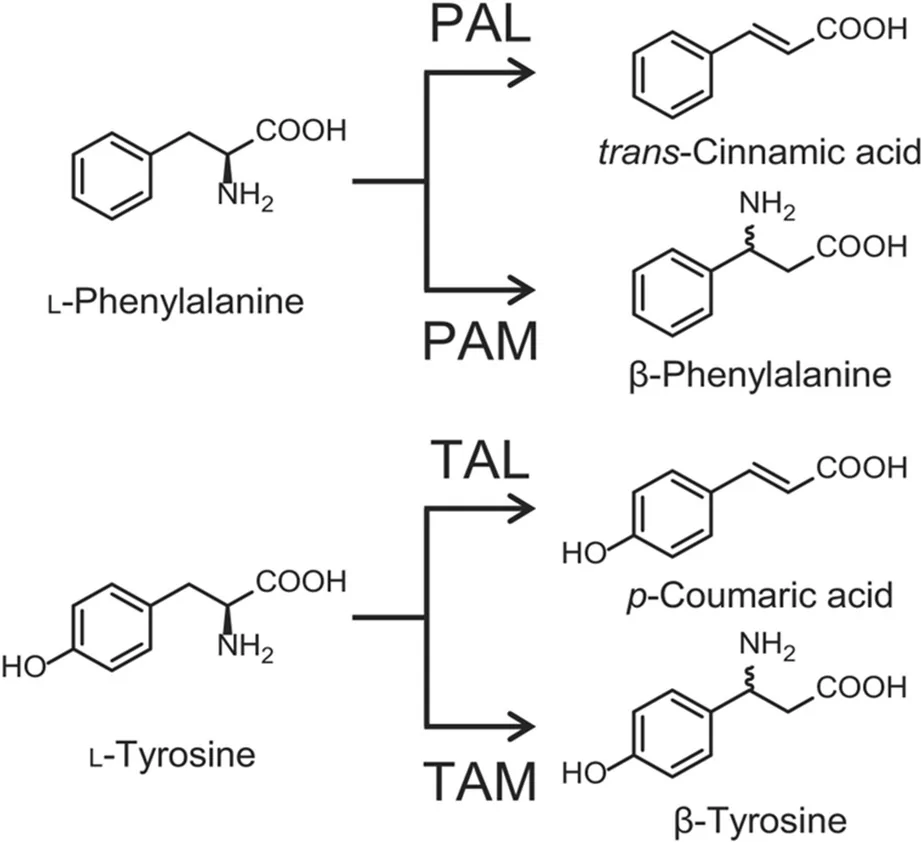
Scheme of the PAL, PAM, TAL and TAM pathways. The lyases release the amino group as ammonia; the aminomutases relocate it to the β‐carbon. From Sakamoto et al. (2026). PAL, phenylalanine ammonia‐lyase; TAL, tyrosine ammonia‐lyase; TAM, tyrosine aminomutase.

Screening 22 grass species, the authors detected appreciable β‐tyrosine in only two: oat, at 49 nmol g^−1^ fresh weight, and maize, at 39 nmol g^−1^. Wheat, barley and sorghum contained barely a trace. Because oat and maize belong to the distantly related BOP and PACMAD clades, respectively, β‐tyrosine production appears scattered across the grass family rather than confined to a single lineage.

The evidence for aminomutase chemistry in maize is elegantly direct. The authors fed maize seedlings tyrosine carrying heavy carbon and nitrogen labels and recovered both labels in the β‐tyrosine produced. The signal for the product retaining the heavy nitrogen was 3.6 times that for the corresponding carbon‐labelled product lacking it. A pathway in which tyrosine was first deaminated and later reaminated would have lost the original nitrogen; its retention therefore strongly supports a direct TAM route.

A homology search identified Zm00001d033286, itself annotated as a PAL, as the maize protein most similar to OsTAM1, at 88% amino acid identity. It was also the only maize PAL or PTAL to carry tyrosine and asparagine at positions Y128 and N449, corresponding to the Y125 and N446 residues known to be important for TAM activity in the rice enzyme. Transient expression in *Nicotiana benthamiana* produced (R)‐β‐tyrosine, accompanied by the same minor (S) component found in maize extracts, where the enantiomeric excess in favour of the (R)‐form is 98%. The authors accordingly renamed the protein ZmTAM1.

Replacing Y128 with the histidine found in PTALs sharply reduced β‐tyrosine production, while replacing N449 with the corresponding lysine abolished it, as did disruption of the MIO binding motif. *In vitro* assays with purified enzyme then gave the most striking result: ZmTAM1 accepted both tyrosine and phenylalanine and exhibited TAM, TAL and PAL activities. Catalytic efficiency was highest for the TAM reaction, followed by PAL and TAL, while no phenylalanine aminomutase activity was detected under the assay conditions.

Why, then, does the enzyme relocate the amino group of tyrosine but release that of phenylalanine? Drawing on work on sorghum PTAL (Jun et al., 2018), Sakamoto proposes that phenylalanine follows a Friedel–Crafts‐type route in which ammonia escapes from the active site. Tyrosine, by contrast, may follow an E2 route that retains the nitrogen long enough for it to be transferred to the β‐carbon. This mechanistic compatibility may explain how one scaffold supports three reactions: as Sakamoto puts it, aminomutase chemistry ‘can be viewed as an extension of the ammonia‐lyase reaction’.

The phylogeny places the TAM lineage within the PAL rather than the PTAL clade, suggesting that the ability to use tyrosine evolved independently in PTALs and TAMs. The split between the PAL and TAM lineages probably predates the divergence of the BOP and PACMAD grasses, although whether their common ancestor already possessed TAM activity remains unresolved. No sequences from *Brachypodium*, wheat, barley, *Miscanthus* or foxtail millet fell within the TAM clade, a pattern consistent with repeated gene loss. Sakamoto suggests that this may reflect a metabolic trade‐off: aromatic amino acids are costly to produce, so TAM may have been lost where the benefit of β‐tyrosine ‘did not outweigh its metabolic cost’.

Sequence alone, however, cannot provide the whole answer. The sorghum protein Sobic.001G160500 and the rice protein LOC_Os11g48110 sit within the TAM clade and carry the same diagnostic Tyr and Asn residues. Nevertheless, sorghum does not accumulate appreciable β‐tyrosine, and the LOC_Os11g48110 protein was previously shown to lack TAM activity (Yan et al., 2015). The two residues are therefore important but not sufficient on their own, leaving other structural features, expression patterns or both to be identified.

The possible biological function of β‐tyrosine is equally intriguing. In laboratory bioassays, 40 μM (R)‐β‐tyrosine reduced *S. hermonthica* radicle elongation by 17% (Sakamoto et al., 2026). This concentration approximated that estimated in jasmonate‐treated maize roots, although the amount present during an actual infection remains unknown. Moreover, maize did not secrete detectable β‐tyrosine into the culture medium, making protection before attachment unlikely. The important test will therefore be whether the compound affects *Striga* during or after formation of the haustorium through which the parasite penetrates its host.

Oat presents perhaps the most tantalising unresolved question. It was the highest β‐tyrosine accumulator in the survey, yet no sequence carrying the diagnostic residues could be found in the available oat resources. Sakamoto suspects that the oat enzyme ‘may still be unannotated, which could explain why it was not detected by amino acid sequence searches’. The metabolite is present in abundance; the gene responsible for it remains to be catalogued.

For application, Sakamoto considers breeding more promising than applying β‐tyrosine as an external compound, particularly because maize already produces it. He remains cautious: increasing β‐tyrosine alone may not provide complete protection, and its effect on infection in living maize plants has yet to be demonstrated. Nevertheless, strengthening an existing chemical defence could offer a new route towards resistance to parasitic weeds.

The paper therefore closes where it began, with the limitations of annotation. Two plant TAMs have now been discovered within the PAL family, both hidden behind PAL labels, while the oat metabolic data suggests that at least one more remains unannotated. The genes may already be in the reference genomes; recognising what they actually do will require experimentation.

## References

[tpj71122-bib-0001] Barros, J. , Serrani‐Yarce, J.C. , Chen, F. , Baxter, D. , Venables, B.J. & Dixon, R.A. (2016) Role of bifunctional ammonia‐lyase in grass cell wall biosynthesis. Nature Plants, 2, 16050. 10.1038/nplants.2016.50 27255834

[tpj71122-bib-0002] Christenson, S.D. , Liu, W. , Toney, M.D. & Shen, B. (2003) A novel 4‐methylideneimidazole‐5‐one‐containing tyrosine aminomutase in enediyne antitumor antibiotic C‐1027 biosynthesis. Journal of the American Chemical Society, 125, 6062–6063. 10.1021/ja034609m 12785829

[tpj71122-bib-0003] Jun, S.‐Y. , Sattler, S.A. , Cortez, G.S. , Vermerris, W. , Sattler, S.E. & Kang, C. (2018) Biochemical and structural analysis of substrate specificity of a phenylalanine ammonia‐lyase. Plant Physiology, 176, 1452–1468. 10.1104/pp.17.01608 PMC5813539 29196539

[tpj71122-bib-0004] Sakamoto, S. , Kobayashi, M. , Yoshikawa, T. , Munakata, R. , Yoshinaga, N. , Okazawa, A. et al. (2026) Maize phenylalanine ammonia‐lyase produces (*R*)‐β‐tyrosine, a growth inhibitor for the parasitic weed *Striga hermonthica* . The Plant Journal, 127, e71046. 10.1111/tpj.71046 PMC13387812 42480067

[tpj71122-bib-0005] Yan, J. , Aboshi, T. , Teraishi, M. , Strickler, S.R. , Spindel, J.E. , Tung, C.‐W. et al. (2015) The tyrosine aminomutase TAM1 is required for β‐tyrosine biosynthesis in rice. The Plant Cell, 27, 1265–1278. 10.1105/tpc.15.00058 PMC4558700 25901084

[tpj71122-bib-0006] Zhang, X. & Liu, C.‐J. (2015) Multifaceted regulations of gateway enzyme phenylalanine ammonia‐lyase in the biosynthesis of phenylpropanoids. Molecular Plant, 8, 17–27. 10.1016/j.molp.2014.11.001 25578269

